# Behaviour of free ranging wild boar towards their dead fellows: potential implications for the transmission of African swine fever

**DOI:** 10.1098/rsos.170054

**Published:** 2017-05-31

**Authors:** Carolina Probst, Anja Globig, Bent Knoll, Franz J. Conraths, Klaus Depner

**Affiliations:** 1 Friedrich-Loeffler-Institut, Federal Research Institute for Animal Health, Institute of Epidemiology, Südufer 10, 17493 Greifswald-Insel Riems, Germany; 2 Universitäts- und Hansestadt Greifswald, Markt 15, 17489 Greifswald, Germany

**Keywords:** wild boar, cannibalism, scavenging, African swine fever

## Abstract

The behaviour of free ranging wild boar (*Sus scrofa*) towards carcasses of their conspecifics potentially infected with African swine fever (ASF) may significantly influence the course of an ASF epidemic. This study aims to better understand the behaviour of wild boar towards their dead fellows. Thirty-two wild boar carcasses on nine study sites in northeast Germany were monitored under field conditions by photo-trapping from October 2015 until October 2016. During this period, a total of 122 160 pictures were taken, thereof 16 111 pictures of wild boar. In both winter and summer, wild boar seemed to be particularly interested in the soil next to and underneath the carcasses. About one third of the visits of wild boar led to direct contact with dead conspecifics. The contacts consisted mostly in sniffing and poking on the carcass. Under the given ecological and climatic conditions, there was no evidence for intra-species scavenging. However, piglets were observed several times chewing bare bones once skeletonization of the carcasses was complete. It must be assumed that all these types of contact may represent a risk of transmission. Both the high tenacity of ASF virus and the long time wild boar carcasses can remain in the environment, allow the persistence of the virus for several months or even years. We therefore consider the rapid detection and removal (or destruction on the spot) of contaminated carcasses as an important control measure against ASF in wild boar.

## 1. Background

African swine fever (ASF) is a notifiable viral disease of domestic and feral pigs (*Sus scrofa*), and is a major threat to animal health and trade for many European countries [1]. An ASF epidemic currently occurs in northeastern parts of the European Union and has spread locally in the wild boar population. Since the detection of the first ASF case in wild boar in Lithuania in late January 2014, more than 4180 cases have been reported in the Baltic States (Estonia, Latvia, Lithuania) and Poland [2]. Most ASF positive wild boar were not detected by active surveillance (hunted), but by passive surveillance (animals found dead) [3]. Initially, two main scenarios were forecasted: due to the high virulence of the virus, ASF could spread so rapidly that it would quickly exhaust the susceptible population and then fade out, or it could cause an epidemic wave that rapidly moves westwards. Three years later, none of the hypotheses proved true; neither did the virus fade out nor did it produce a fast epidemic wave. Rather, the disease has been slowly spreading westwards. The main problem seems to be the long persistence of the virus in contaminated carcasses, which may remain in fields or forests for weeks [3–5]. The risk of ASF introduction into Germany is estimated as high [6,7]. For the spread of ASF into free areas, direct contact with contaminated carcasses is regarded as one of the most important risk factors [8–10].

African swine fever virus (ASFV) is extremely stable in the environment and is efficiently transmitted via blood and meat of infected animals. It can persist at 4°C for over a year in blood, several months in boned meat and several years in frozen carcasses [11,12]. The virus also survives the process of putrefaction [13]. Due to its high tenacity, the spread of ASFV through carcasses is considered to be more important than direct contact with live infectious animals, depending on the frequency, at which naive animals have contact with infected carcasses within their range of daily movements [13–17].

In contrast to the characteristics of ASFV, little is known about the behaviour of free ranging wild boar towards their dead fellows. To our knowledge, only few studies have examined the factors governing carrion use by vertebrates [18–21] and none of them explicitly focused on interaction patterns, the frequency and intensity of contacts, potential cannibalism and the conditions that may trigger these phenomena among wild boar and carcasses of their conspecifics. However, these data are of particular interest for understanding the persistence and spread of ASF among European wild boar, which represents a new population host since the emergence of ASF in Eurasia. They are also required for fine-tuning theoretical models that estimate ASF transmission [22–25]. The aim of this study is to provide field data on the interfaces between live wild boar and wild boar carcasses to better understand the dynamics of ASF perpetuation in a wild boar population. The results might be of particular interest for countries with a high wild boar density such as Germany [26,27], where ASF has never occurred before.

## 2. Methods

### 2.1. Study sites

**Figure 1.**
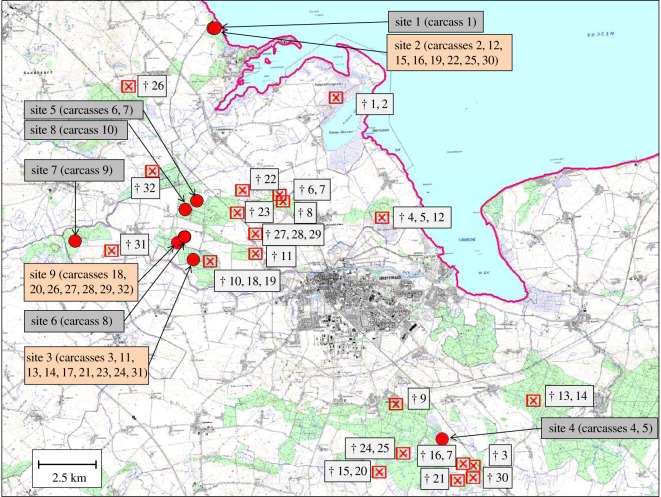
Localization of the sites of death of the 32 wild boar and the nine study sites (eight winter sites in grey, three summer sites in orange).

The study area was located in a hunting ground comprising 9000 hectares around the town of Greifswald, northeast Germany (54°6′ N, 13°23′ E). The landscape is dominated by agriculture, forestry and a low number of human settlements (figure 1). The study was conducted in nine different sites in five independent, mixed coniferous/deciduous forests dominated by oak (*Quercus robur*), alder (*Alnus glutinosa*) and beech (*Fagus sylvatica*). The forests are surrounded by crop production fields, where mainly wheat, maize and oil-seed rape are grown. The climate is influenced by the Baltic Sea. The cold season (December to March) usually covers periods with snow and temperatures below zero. Within the ground of 9000 hectares, the hunting bags 1995–2005 and 2005–2015 were 1547 and 2598 wild boar, which corresponds to 0.17 and 0.29 wild boar per hectare, respectively. The hunting bag in winter 2015/2016 amounted to 345 wild boar (Paul Däubner 2016, personal communication). In addition to wild boar, there are three species of ungulates in the area: Red deer (*Cervus elaphus*), fallow deer (*Dama dama*) and roe deer (*Capreolus capreolus*). Also, numerous species of carnivores and carrion-eating birds are present.

Nine study sites in five different forests were used to monitor the carcasses, eight in winter and three in summer (figure 1). Sites 1 and 4–8 were used only in winter; sites 2 and 3 were used in winter and summer, while site 9 was only used in summer.

**Figure 2.**
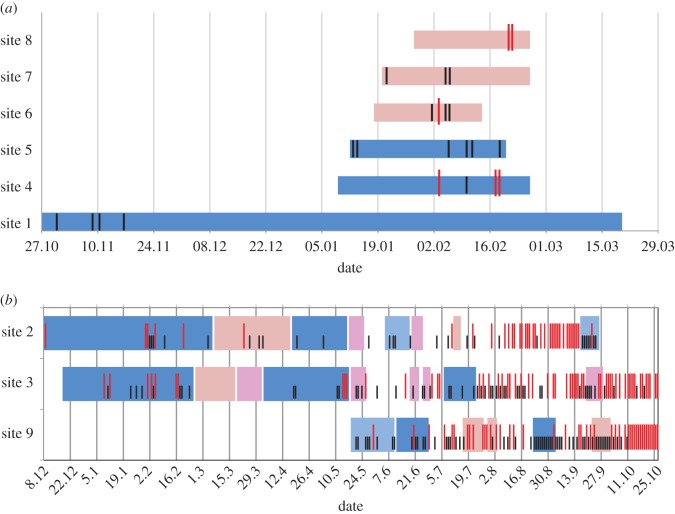
Timeline of monitoring wild boar carcasses. Each bar represents one carcass; the length of the bar indicates how long it took until skeletonization of the carcass was complete. Bars in dark blue = adult male; light blue = young male; dark pink = adult female; light pink = piglets. Days/nights with wild boar visits are plotted with red (direct contact) and black (no direct contact) lines. (*a*) Monitoring individual carcasses in winter (sites 1, 4, 5, 6, 7 and 8). (*b*) Consecutive monitoring of wild boar carcasses in winter and summer (sites 2 and 3) and in summer (site 9).

On sites 1, 6, 7 and 8, we placed one carcass and on sites 4 and 5 two piglets at the same time (figure 2*a*). Sites 2, 3 and 9 were consecutively used to place several carcasses there; new carcasses were only exposed after the skeletonization of the previous carcass was complete (figure 2*b*). Sites 2, 3 and 9 were continuously monitored starting with the exposure of the first carcass until the end of the study on 27 October 2016. On site 2, we placed a total of eight carcasses; on site 3, nine, and on site 9 seven carcasses (thereof two piglets at the same time).

All sites were located in places where field signs indicated the presence of wild boar, i.e. fresh tracks, rooting, nests and diggings. Aerial distances between the study sites ranged between 20 m (sites 1 and 2) and 18 km (sites 1 and 4). During the whole study period, no hunting took place in the surroundings of approx. 1 km. Although winter feeding is regularly provided in the surrounding area, no additional feeding was provided in the vicinity of the study sites, with the exception of site 9, where 500 grams of maize per day was provided through an automatic dispenser approximately 10 m apart from the carcass site in July and August 2016. Sites 1, 2, 3 and 6 were baited with beech tar on one tree to attract wild boar. Sites 2 and 3 were mown with a scythe when necessary for a free view on the carcass in summer.

### 2.2. Carcasses

Thirty-two wild boar carcasses of various origins (hunted, traffic accidents) and of different age and gender (six adult male, five young male, eight females, 13 piglets) were used (table 1). The carcasses were acquired from local hunters, who estimated their initial body mass and age. Carcasses were either exposed freshly or kept cool in a refrigerator until placed on site, either complete or eviscerated. They were placed directly on the forest ground, so that terrestrial and avian scavengers had unrestricted access. If shot, they were placed with the bullet hole underside. Small carcasses were tied up with a rope to avoid them being dragged out of the scope of the camera by scavengers. Moreover, five carcasses of ruminants (three adult roe deer, one red deer calf, and one young male red deer) and the viscera of one fallow deer were used as ‘controls’ at sites 2, 3, 6, 8 or 9 respectively. The ‘controls’ were placed 1 m (sites 2, 3, 6, 8) or 30 m (site 9) apart from the wild boar carcasses.

**Table 1.** Details on the carcasses used in the study.

site	carcass	age class, gender, body weight	date of exposure	cause of death	carcass state	skeletonization completed
1	1^a^	adult male, 100 kg	27 Oct 2015	shot	whole	early March
2	2	adult male, 60 kg	08 Dec 2015	shot	only jaws cut off	end of January
3	3^b^	adult male, 80 kg	18 Dec 2016	fallen tree	only head cut off	early February
4	4,5	piglets, 2 and 12 kg	09 Jan 2016	hunted	eviscerated	mid-February
5	6,7	male piglets, 10 kg	14 Jan 2016	road kill	eviscerated	early March (removed by scavengers)
6	8^c^	piglet female, 30 kg	18 Jan 2016	road kill	eviscerated	mid-February (removed by scavengers)
7	9	young female, 40 kg	20 Jan 2016	hunted	eviscerated	early March
8	10^d^	piglet, 15 kg	28 Jan 2016	hunted	eviscerated	mid-February
3	11^e^	piglet, 20 kg	25 Feb 2016	shot	eviscerated	mid-March
2	12^f^	piglet, 20 kg	07 Mar 2016	shot	eviscerated	early April
3	13	juvenile, 35 kg	18 Mar 2016	road kill	whole	early May
3	14	adult male, 80 kg	01 Apr 2016	road kill	whole	end of May
2	15	adult male, 70 kg	17 Apr 2016	shot	eviscerated	end of May
2	16	young female, 28 kg	17 May 2016	shot	eviscerated	mid-May
3	17	young female, 26 kg	17 May 2016	shot	eviscerated	end of May
9	18	young male, 35 kg	18 May 2016	shot	whole	early June
2	19	young male, 30 kg	05 June 2016	shot	whole	mid-June
9	20	adult male, 62 kg	07 June 2016	shot	eviscerated	mid-June
3	21	young female, 40 kg	17 June 2016	shot	whole	mid-June (removed by scavengers)
2	22	young female, 50 kg	19 June 2016	shot	eviscerated	end of June
3	23	young female, 25 kg	24 June 2016	shot	eviscerated	end of June
3	24	young male, 80 kg	05 July 2016	shot	whole	mid-July
2	25	male piglet, 17 kg	11 July 2016	shot	whole	mid-July
9	26	male piglet, 8 kg	18 July 2016	shot	eviscerated	mid-July
9	27,28	female piglets, 8 kg	25 July 2016	shot	eviscerated	end of July
9	29^g^	young male, 80 kg	19 Aug 2016	shot	whole	end of August
2	30	young male, 47 kg	16 Sep 2016	shot	eviscerated	end of September
3	31	young female, 60 kg	18 Sep 2016	shot	whole	end of September
9	32	female piglet, 25 kg	22 Sep 2016	shot	whole	end of September

^a^By the end of November the carcass lay almost completely in the water.

^b^Carcass was laid beside the carcass of a red deer calf.

^c^Carcass was placed beside the carcass of a red deer, who had probably broken his leg.

^d^Carcass was placed beside the fresh viscera of a fallow deer.

^e^Carcass was placed beside the fresh carcass of a fallow deer.

^f^Carcass was placed beside the fresh carcass of a roe deer.

^g^The fresh carcass of a roe deer was exposed approximately 30 m apart.

### 2.3. Cameras

Each carcass was monitored either by an infrared heat- or a motion-sensitive digital camera. We used five different game camera models, namely Seissiger Special-Cam 3 Classic (Anton Seissiger GmbH, Würzburg, Germany), Maginon WK 3 HD (Supra, Kaiserslautern, Germany), Dörr Snapshot UV555 (Dörr GmbH, Neu-Ulm, Germany), Moultry A5 and Moultry I40 (Moultrie Alabaster, USA) (table 2). During the first weeks (site 1), we obtained short video footages; however, wild boar approached the sites only in the dark, and the quality of the videos did not allow accurate assessment of the wild boar behaviour, despite using the best possible camera set-up. In an attempt to overcome this, we switched to photos in mid-November 2015. The cameras were on standby mode during 24 h a day and set to take one photo every 1–10 s if activated. They were installed on trees at a distance of 4–8 m to the carcass and a height of 1–2.2 m above the ground. After the camera originally installed at site 2 had failed several times, we replaced it with another camera model (this one was considered as ‘primary camera’) in late March 2016. During winter, we used one camera on each site. However, we realized that wild boar were often present without moving into the scope of the cameras. We therefore installed in summer one additional camera at site 2 and two additional cameras at site 3, focusing on the carcass sites from different angles (table 2).

**Table 2.** Technical specifications of the digital cameras used in the study.

camera model (*) confirmatory camera	mode	shot frequency setting	site (carcasses monitored)
Seissiger Special-Cam 3 Classic	motion sensitive	every 1 s	Site 1 (1);
			Site 2 (12;15;16;19;22;25;30)
			Site 5 (6,7)
			Site 8 (10)
			Site 9 (18; 20; 26; 27; 28; 29; 32)
Maginon WK 3 HD	heat sensitive	every 6 s	Site 2 (2);
			Site 4 (4,5)
			Site 6 (8)
			Site 7 (9)
Moultry I40	motion sensitive	every 10 s	Site 3 (3;11;13;14;17;21;23;24;31)
Moultry A5 (*)	motion sensitive	every 10 s	Site 2 (25;30)
			Site 3 (14;17;21;23;24;31)
Dörr Snapshot UV555 (*)	motion sensitive	every 5 s 3 shots per motion	Site 3 (23;24;31)

All cameras were directed and rearranged as necessary to keep the carcasses in view and to capture all movements and animal activities in a radius of approximately 4–10 m around the carcasses. Date, time and temperature were recorded automatically on each picture.

Every 2–14 days, the carcasses and the cameras were inspected and the presence of wild boar in the surroundings was checked by searching for fresh tracks and additional photo-trapping 30–80 m away from sites 2, 3 and 9 (pictures not included in the analysis). During each visit, pictures were taken with a handheld digital camera (Canon Power Shot A 3400) to document the status of carcass decomposition. Monitoring the sites was stopped when all edible biomass of the carcass had been consumed (only dried out skin and bones, completely clean of soft tissue, were left over) or when the remains had been taken away by scavengers.

### 2.4. Analysis

The study period was divided into two seasons, winter (27 October 2015–30 April 2016) and summer (1 May–27 October 2016). Image analysis and quantification was performed in the following way:

First, we screened all pictures and counted those that displayed at least one identifiable animal. On several occasions, the cameras took pictures without any animal visible on them, e.g. due to moving branches, or the animal could not be identified due to fog, lack of light, or other reasons. These pictures were defined as ‘not evaluable’ and excluded from further analysis. All eligible pictures showing wild boar were selected and saved in a separate folder, one for each study site and camera. Although the focus of this study was put on wild boar, we also analysed the visit frequency of other animals. However, to enable comparison between images from different sites and seasons regarding ‘other species than wild boar’, we only analysed the pictures of the cameras that had first been installed, i.e. one camera per site (called ‘primary camera’). Regarding wild boar, we analysed all pictures taken with both ‘primary’ and ‘confirmatory’ cameras.

In a second step, all wild boar pictures were analysed separately including date (days after exposure), start time, end time (time spent on site), the number of pictures, the number of animals simultaneously present, and the minimum distance between wild boar and carcass.

The third step was performed on a television screen with the help of one to three hunters from the respective area. All images that showed direct contact with the carcass or its remnants were analysed in detail to determine the behaviour of the animals (sniffing, poking, moving bones aside to get access to the soil underneath, etc.). In the analysis, we considered touching with the snout the decomposing remains of a carcass as direct contact, and feeding on carrion of the same species as intra-species scavenging.

Excel (Microsoft, Redmond, USA) was used for data recording and analysis.

## 3. Results

The trial encompassed a total of 367 days and nights starting from 27 October 2015 until 27 October 2016. During this period, we performed a total of 150 on-site inspections (100 in winter, 50 in summer). The total sampling effort was 1136 camera-days (number of days all cameras were in use) yielding a total of 122 160 pictures, thereof 21 567 in winter and 110 593 in summer. Depending on the temperature and the size of the carcass, it took between 4 days (young female in summer) and three months (adult male boar sunken in a wallow in winter, electronic supplementary material, figure S8) until skeletonization was complete.

### 3.1. Observed species

The primary cameras yielded a total of 40 141 pictures (19 590 in winter and 20 551 in summer), of which 36 594 (91%) were evaluable, i.e. they showed at least one identifiable animal (table 3). Of the evaluable pictures, 14 743 (40%) displayed carnivores, 12 063 (33%) birds, 9451 (26%) wild boar, and 337 (1%) other species.

**Table 3.** Number of visits and pictures, including those that document direct contact between wild boar and the carcass.

		visits			pictures							
site	carcass number	number of *visits*	number of *nights* with visit	number of *visits* with direct *contact*	number of pictures primary camera	total number of pictures	number of pictures with *visit* primary camera	total number of pictures with *visit*	number of pictures with *contact* original camera	total number of pictures with *contact*	percentage pictures with *contact* original camera	percentage total pictures with *contact*
1	1	4	4	0	3525	3525	16	16	0	0	0	0
2 winter	2,12,15	15	15	6	3223	4186	170	170	42	42	19	25
2 summer	16,19,22,25,30	74	50	41	1890	5484	1266	3091	230	1213	18	39
3 winter	3,11,13,14	23	17	8	6405	7419	141	153	19	19	13	12
3 summer	17,21,23,24,31	154	90	71	3256	79 704	654	5561	236	1954	35	35
4	4,5	4	4	3	378	378	80	80	16	16	20	20
5	6,7	6	6	0	892	892	49	49	2	2	4	4
6	8	5	4	1	840	840	50	50	1	1	2	2
7	9	3	3	0	1122	1122	48	48	0	0	0	0
8	10	3	2	2	3205	3205	63	63	12	12	19	19
9	18,20,26,27,28,29,32	229	103	57	15 405	15 405	6830	6830	1455	1455	21	21
*total winter*		*63*	*55*	*20*	*19 590*	*21 567*	*617*	*629*	*92*	*92*	*15*	*15*
*total summer*		*457*	*243*	*169*	*20 551*	*1 00 593*	*8750*	*15 482*	*1921*	*4622*	*22*	*30*
*total*	* *	*520*	*298*	*189*	*40 141*	*1 22 160*	*9367*	*16 111*	*2013*	*4714*	*21*	*29*

Altogether, 23 animal species were identified at the study sites: thirteen species of mammals, including wild boar, red fox (*Vulpes vulpes*), European pine marten (*Martes martes*), polecat (*Mustela putorius*), raccoon dog (*Nyctereutes procyonoides*), raccoon (*Procyon lotor*), European badger (*Meles meles*), European otter (*Lutra lutra*), red deer, roe deer, water vole (*Arvicola terrestris*), red squirrel (*Sciurus vulgaris*), and domestic dog (*Canis familiaris*), and 10 bird species, including common raven (*Corvus corax*), common buzzard (*Buteo buteo*), white-tailed sea eagle (*Haliaeetus albicilla*), red kite (*Milvus milvus*), hooded crow (*Corvux cornix*), common blackbird (*Turdus merula*), European robin (*Erithacus rubecula*), great tit (*Parus major*), wood nuthatch (*Sitta europaea*) and common starling (*Sturnus vulgaris*).

Three mammal and six bird species were observed scavenging on carcasses in different decomposition stages, namely foxes, pine martens, raccoon dogs, ravens, buzzards, sea eagles, kites, crows and starlings. The most readily attracted scavengers were birds, in particular common buzzards and ravens.

During winter, birds visited the carcasses more frequently (66% of the pictures) than mammals (34% of the pictures). However, foxes and raccoon dogs were responsible for most consumption of the carrion.

In summer, almost exclusively mammals (98%) approached the carcasses, mainly raccoon dogs (78% of the pictures with carnivores). However, during the warm season, adult carrion flies and beetles started to lay eggs on the carcasses a few hours after exposure. Subsequently, large part of the carcasses was not consumed by mammals or birds, but rapidly metabolized by insect larvae.

On several occasions, the carcasses were visited by more than one species simultaneously, e.g. buzzard and raven or fox and marten. Wild boar were not involved in mixed-species encounters.

### 3.2. Wild boar

All study sites were approached by wild boar. Fresh tracks or additional photo-trapping demonstrated that they were also regularly present in the vicinity of the sites, even if they did not come close enough to enter the scope of the cameras.

A total of 520 wild boar visits were recorded in 298 days/nights (26% of all 1136 camera-nights), thereof 63 visits (12%) in 55 nights in winter and 457 visits (88%) in 243 days/nights in summer (table 3). In most days/nights (191; 64%), only one visit was observed. In 107 days/nights (36%), two or more visits were observed: in 59 days/nights two visits, in 37 days/nights three, in 8 days/nights five, in 2 days/nights six, and in 1 day/night nine.

Most visits were observed in September (157) and October (107), while in November, December, March and April only 3, 1, 4 and 4 visits were recorded, respectively. The 520 visits were documented with a total of 16 111 pictures, thereof 629 (4%) in winter and 15 482 (96%) in summer. This means that 13% of all 122 160 pictures recorded at least one wild boar.

More than half of the 16 111 pictures with wild boar were taken by the primary cameras (9367 pictures, 58%), thereof 617 in winter, and 8750 in summer (table 3). This means that 23% of the pictures of the primary cameras recorded at least one wild boar (3% in winter, 43% in summer).

The three confirmatory cameras provided 6744 additional pictures of wild boar (42%). The confirmatory camera at site 2 took 1825 pictures, but there was no evidence for additional visits not recorded by the primary camera. The two confirmatory cameras at site 3 took 4907 pictures and provided evidence for another 69 visits (883 pictures), which had not been captured by the primary camera.

The animals approached the site alone (adult or young pigs), as part of small groups, or as part of large routs with up to 17 animals (mainly sows with piglets). The average group size was 4.1 (standard deviation 3.1) and ranged from 1 (site 1) to 4.5 (site 4).

After exposure of a fresh carcass, the sites were approached for the first time on average on day 7 (between day 0 and 25). Direct contact with a carcass occurred on average on day 15 (between day 1 and 32). Direct contact was observed at 16 carcasses (counting piglets 4/5 and 27/28 as one) (figure 2*a*,*b*). Contact in a fresh stage of decomposition was observed only at carcass 2 (day 1). Contact in the bloat stage (inflation of the abdomen) was observed at carcasses 26, 32 (day 2) and 27/28 (day 3). All other carcasses were only touched in a more advanced stage of decay. In seven cases (carcasses 17, 20, 22, 23, 24, 25, 29), contact took place after skeletonization was already complete (figure 2*b*). The longest period of time between deposition of the carcass and the first contact was observed on carcass 14, which was first touched only on day 43, while carcasses 11 and 13 were not approached at all (although wild boar had earlier been observed at carcass 3 and later at carcass 14, which were exposed at the same site).

The average minimum distance kept to the carcass by the wild boar that came closest on each visit respectively ranged between 30 cm (site 8) and 1.8 m (site 9) (average 1.5 m; standard deviation 1.6). The average time a group or a solitary animal spent at the site during each visit ranged from 0.5 (site 8) to 7.2 min (site 7) (average 5.5 min; standard deviation 6.4).

The most frequent behaviour documented was rooting in the surroundings of the carcass (electronic supplementary material, figure S1). As long as the carcasses were fresh or (in summer) covered by maggots, wild boar often approached the site, stopped, looked for a few seconds at the carcasses, and then turned and walked away without touching them (electronic supplementary material, figure S2). Especially young animals displayed obvious signs of excitement (e.g. bristling neck hairs).

Direct contact of at least one wild boar with a carcass was observed at all sites. Direct contact occurred in a total of 189 visits (36% of all 520 visits) in 119 nights, thereof 20 visits in winter (32% of the visits) and 169 visits in summer (37% of the visits). Most direct contacts were observed in August (33), September (52) and October (54). Direct contacts are documented with a total of 4714 pictures (29% of all 16 111 pictures with a visit) (table 3).

Almost half of the 4714 pictures with direct contact were taken by the primary cameras (2013 pictures, 43%), thereof 92 in winter, and 1921 in summer. This means that in 21% of the 9367 pictures with visits from the primary cameras, direct contact is documented.

The closest type of contacts consisted in sniffing and poking on the carcass (without leaving any signs of cannibalism, e.g. bite marks), chewing on bare ribs (in summer on all three sites), and in rooting on the soft soil that had formed after decomposition of several carcasses on the same spot (electronic supplementary material, figure S3). On 15 occasions, a wild boar was observed taking a rib into the mouth and chewing on it (72 pictures) (electronic supplementary material, figure S4). On 5 occasions (39 pictures), a wild boar was observed rooting between the putrefying leftovers of a carcass, and on 2 occasions (12 pictures) a wild boar was observed rolling on the soft ground on site 3 where the remains of a carcass were rotting (electronic supplementary material, figure S5). One young male was observed pushing carcass number 3 (adult male, site 3) aside and rooting in the soil underneath it, with the consequence that the ground around the carcass was completely stirred up on day 50 (electronic supplementary material, figure S6). This phenomenon was observed in a similar way at sites 2 and 9 as well: wild boar pushed larger bones (skull, scapulae) aside while rooting in the soil underneath. On two occasions, wild boar came into contact with the carcass apparently by chance while feeding on carrion of a wild ruminant placed 1 m beside (electronic supplementary material, figure S7).

The ruminants used as ‘controls’ at sites 2 and 3 were consumed by wild boar within a few days. Almost all the edible portions of the carcasses were consumed, including large bones. While feeding on ruminants, wild boar were apparently not interested in their dead fellows, even if they were in closest proximity. The red deer on site 6 was almost entirely consumed by foxes and avian scavengers.

During the study period, the monthly mean temperature was 7.3°C (November 2015), 6.7°C (December 2015), −0.8°C (January 2016), 3.4°C (February), 4.3°C (March), 7.8°C (April), 13.9°C (May), 17.3°C (June), 18.2°C (July), 17.5°C (August), 15.6°C (September), and 8°C (October) respectively, and the monthly rainfall amounted to 82, 42, 40, 47, 26, 23, 20, 52, 49, 38, 69, 31 l m^−2^ (http://www.wetterkontor.de).

## 4. Discussion

Although the underlying question of this study originates from the field of epidemiology (transmission patterns of ASF), the approach was based on ethological methods (camera trapping to observe wild boar behaviour).

During the first eight weeks of the study, we faced failure problems with the first camera. As a consequence, we have no complete photographic documentation of the scavenging activities at this site. Also, our first attempts to take video footages failed due to the poor quality of the videos taken in the dark, which did not allow identifying animals or determining the behaviour with the required accuracy. We therefore decided to rely on pictures rather than on videos. To improve the monitoring of the presence of wild boar, we reduced the number of study sites in summer, but installed additional, ‘confirmatory’ cameras.

For reasons of practicability, we had to use carcasses with many different individual characteristics (e.g. gutted, wounded, complete). On the other hand, by using both male and female and different age groups, we could rule out that cannibalism is avoided only under special circumstances (e.g. due to the odour of adult males or piglets, or hunted animals). On the contrary, age, gender and the smell of the carcasses seemed to have no influence on the behaviour, since all carcasses, including those of adult males, were approached. Equally important, we considered that wild boar have a distinctive social behaviour and might distinguish between members of their own group and dead animals of other groups. Therefore, we placed all carcasses as far as possible away from the place where they were shot or killed, thereby minimizing the probability that they belonged to the group that lived close to the study site. We also selected study sites as far as possible from each other, so that the probability was minimized that the home ranges of the observed animals overlapped.

Wild boar seemed to find carcasses of conspecifics more attractive in summer/fall than in winter, maybe due to increased nutritional requirements in the warm season, which may be related to the rapid growth of piglets and the fact that they are suckling milk at this time of the year. In winter, wild boar were exclusively observed in the dark and not seen returning to the carcass within the same night. In summer, they were seen day and night, and the same groups sometimes returned to the carcass some hours after the previous visit. However, with few exceptions, they only stayed at the carcass site for a short time (less than three minutes), which might be a further indication that they were not interested in scavenging on the carcass. The low number of visits in March and April might be due to the fact that females are busy with building farrowing nests at this time of the year.

Wild boar seemed to avoid direct contact with fresh carcasses; on average, 15 days passed until they had direct contact with a dead conspecific. Taking into account the remarkably long time during which ASFV may remain infectious in a protein-rich environment, these late contacts may be as important as contacts with a fresh carcass with respect to the risk of ASF transmission. Therefore, we believe that even if a carcass is detected and removed several days after the death of the animal, removal is still an effective control measure.

In this study, every third visit has led to direct contact with a dead conspecific. The contact seemed to occur mostly in the context of rooting in the immediate surroundings or by curiosity. Overall, a surprisingly large number of groups and individuals got into direct contact with the carcasses. Especially in late decomposition stages, at first glance, the images often seemed to show intra-species scavenging. However, detailed analysis revealed that wild boar sniffed and poked the carcasses and pushed the leftovers (bones, skin) aside to root and excavate beneath them, but without leaving any sign of scavenging.

The results indicate that under favourable feed conditions and mild climate in Germany, intra-species scavenging among wild boar is not a frequent phenomenon. Indeed, wild boar were observed avidly scavenging on red and roe deer placed beside wild boar carcasses, without coming into contact with the carcasses of their own fellows. Even during a short cold period in January 2016 with temperatures of up to 15 degrees below zero, snow cover and frozen soil, there was no evidence for cannibalism, even piglets exposed in early January stayed untouched. Instead, we found that wild boar carcasses remained present for quite a long time in winter. Especially the skin of adult males seemed to be hard to open. It took, for example, three weeks until buzzards were finally able to tear the first pieces of intestines out of carcass 1. Instead of tearing the skin, they used a small hole in the abdomen to eat from the soft tissues (electronic supplementary material, figure S8). However, once the carcass was open, it was rapidly consumed particularly by avian scavengers and foxes or raccoon dogs, at least during winter, while beetles, carrion flies and their larvae, i.e. maggots, competed with birds and mammalian scavengers for this source of food in summer.

Dietary studies have shown that wild boar are omnivorous and that more than 85% of their diet consists of vegetables. Their food selection depends mainly on energy requirements and is influenced by seasonal and geographical factors [28–30]. The finding that intra-species scavenging among wild boar played hardly any role under the given circumstances is not surprising as the study was carried out in an area where plenty of food is available during all seasons (large forage areas with beechnuts, acorns and other tree seeds, as well as agricultural products like cereals, fruit and vegetables) and during a mild winter. Well-fed wild boar are probably less interested in scavenging on decomposing carcasses of conspecifics. However, the feed-rich situation is very similar across the whole country, so we can assume that the behaviour of wild boar towards their dead conspecifics would be more or less similar in most parts of Germany and central Europe. The potential impact of rough climatic conditions and scarcity of food has to be addressed in other study areas.

ASFV might not only be transmitted through infected carcasses, but also by contact with infectious material like blood or other body fluids [31]. It has been shown that minute amounts of blood from infected pigs can trigger the infection in naive pigs kept in the same stable [17]. Under experimental conditions, wild boar have been observed nibbling on ill and dead stablemates with the consequence of subsequent fatal infection [32]. In this study, piglets were observed several times chewing on ribs of dead wild boar. It is therefore possible that they exhibit the same behaviour more frequently, e.g. when they nibble on smaller bones like vertebrae, but it may not always be possible to observe this by camera trapping. As a consequence, the frequency of direct contact to bones of dead wild boar may be underestimated in this study. Despite putrefaction, ASFV may remain infectious in bone marrow for a long time [33]. It remains open, however, if chewing is sufficient to transmit ASF as long as the bones are not broken and the animals have no contact with bone marrow.

On the three study sites where several carcasses were consecutively placed on the same spot in summer, the by-products of decay led to a wet, dark, loose, soft substrate in the soil. Our findings suggest that wild boar regardless of their age were possibly more interested in this particular soil surrounding and underneath the carcasses than in the carcasses themselves. Previous studies have shown that decomposition of carcasses of large vertebrates returns nutrients (especially phosphorus) to the soil and causes local changes in soil chemistry [34]. In any case, the viral load of the soil underneath and in the vicinity of an ASF contaminated carcass should be further investigated in areas where ASF occurs in wild boar. It is also known that carcasses provide an important food resource for several generations of insects [35,36] and that the micro-fauna gathering around a carcass may also represent attractive food to wild boar [19]. It has therefore been postulated that the higher ASF prevalence observed in the Baltic states in summer may be due to the fact that wild boar acquire the infection via consuming eggs or larvae directly present in large amounts at the carcasses. However, we were not able to detect wild boar feeding on maggots present on or close to carcasses.

We cannot determine whether the observed contacts would have been intensive enough to transmit ASF to a susceptible wild boar. However, if we assume that (i) all 32 carcasses were contaminated with ASFV, (ii) most direct contacts would have been effective in terms of disease transmission, and (iii) all groups that approached the study sites were an epidemiological unit, then most of the wild boar observed in this study would have been exposed to ASFV. The possible efficiency of carcass removal still needs to be investigated. In any case, our results indicate that ASF transmission does not necessarily occur within the first days after the death of an infected wild boar, if mediated through contact with an infected carcass, but rather in a more advanced state of decomposition. However, infection of a single animal may lead to the spread of ASFV to other members of the social group. Therefore, all efforts should be taken to localize and remove wild boar carcasses as soon as possible, although they are not easy to find. If removal is not feasible, the carcass should be treated with strong repellents to prevent wild boar approaching the site.

## 5. Conclusion

Under favourable food conditions, intra-species scavenging among wild boar seems to play no role in the study area. Wild boar rather seemed to be reluctant to directly approach carcasses of their own fellows, especially as long as they were fresh or covered by maggots. Contacts mainly consisted of rooting near or underneath the carcasses, sniffing and poking, and chewing bare bones.

The high tenacity of ASFV and the relatively long time that remnants of dead wild boar may remain in the environment are likely to contribute substantially to the contamination of the habitat (soil underneath the carcasses) and to the presence of infectious ASFV for a long time, perhaps months or even years in a region. We therefore consider the rapid detection and removal (or destruction on the spot) of contaminated carcasses as an effective control measure against ASF transmission in the wild boar population. Hunters should therefore be appropriately trained and involved in ASF contingency measures.

## Supplementary Material

ESM 1 Table - Details on pictures and visits

## Supplementary Material

ESM Figure 1 - Wild boar rooting at site 3

## Supplementary Material

ESM Figure 2 - Wild boar are curious, but do not touch the carcasses

## Supplementary Material

ESM Figure 3 - Wild boar are attracted by the soft ground underneath rotten carcasses

## Supplementary Material

ESM Figure 4 - Wild boar chewing on bones (bare ribs) after skeletonization is complete

## Supplementary Material

ESM Figure 5 - Wild boar rolling on soft ground on site 3

## Supplementary Material

ESM Figure 6 - Ground underneath carcass 3 is stirred up

## Supplementary Material

ESM Figure 7 - Wild boar feeding on wild ruminant and leaving wild boar carcass aside

## Supplementary Material

ESM Figure 8 - Skeletonization process of carcass 1

## Permission to carry out fieldwork

The Department of Forestry of the City of Greifswald as the competent authority participated in the study. The veterinary office of the City of Greifswald, SG Veterinärwesen, Mendelejewweg 16, 17438 Greifswald, was informed about the study through a formal letter on 4 November 2015.

## Data accessibility

The following supporting data is available through https://openagrar.bmel-forschung.de/receive/openagrar_mods_00026443. Electronic supplementary material, data 1—Details on all recorded pictures and wild boar visits (Excel file).
