# Supplementary figures and images for: Behaviour of free ranging wild boar towards their dead fellows: potential implications for the transmission of African swine fever
Source: R Soc Open Sci. 2017 May 31;4(5):170054. doi: 10.1098/rsos.170054 (PMC5451812; doi:10.1098/rsos.170054)

## Slide 1
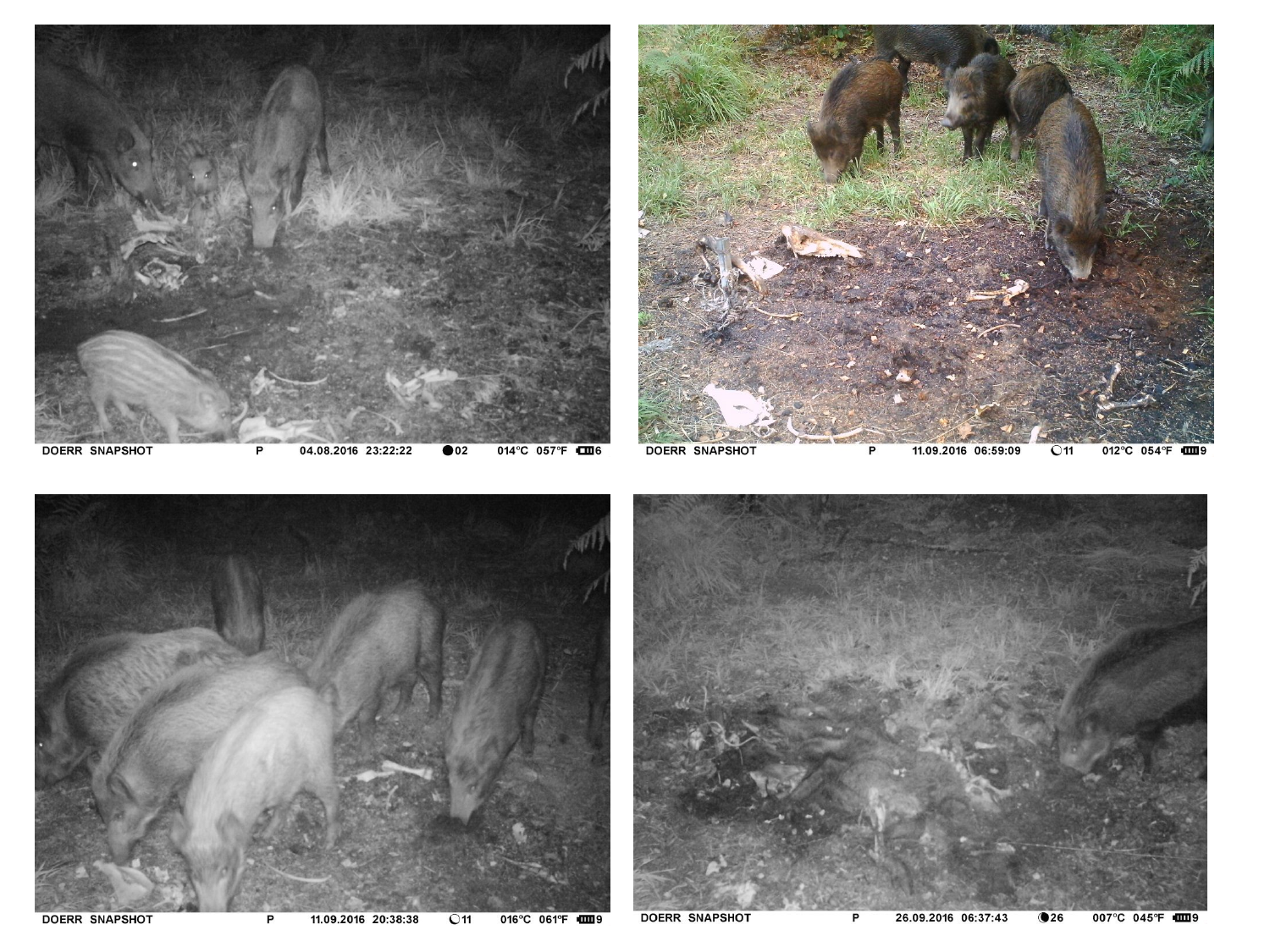

Supplement: ESM Figure 1 - Wild boar rooting at site 3 [file rsos170054supp2.pptx]

## Slide 1
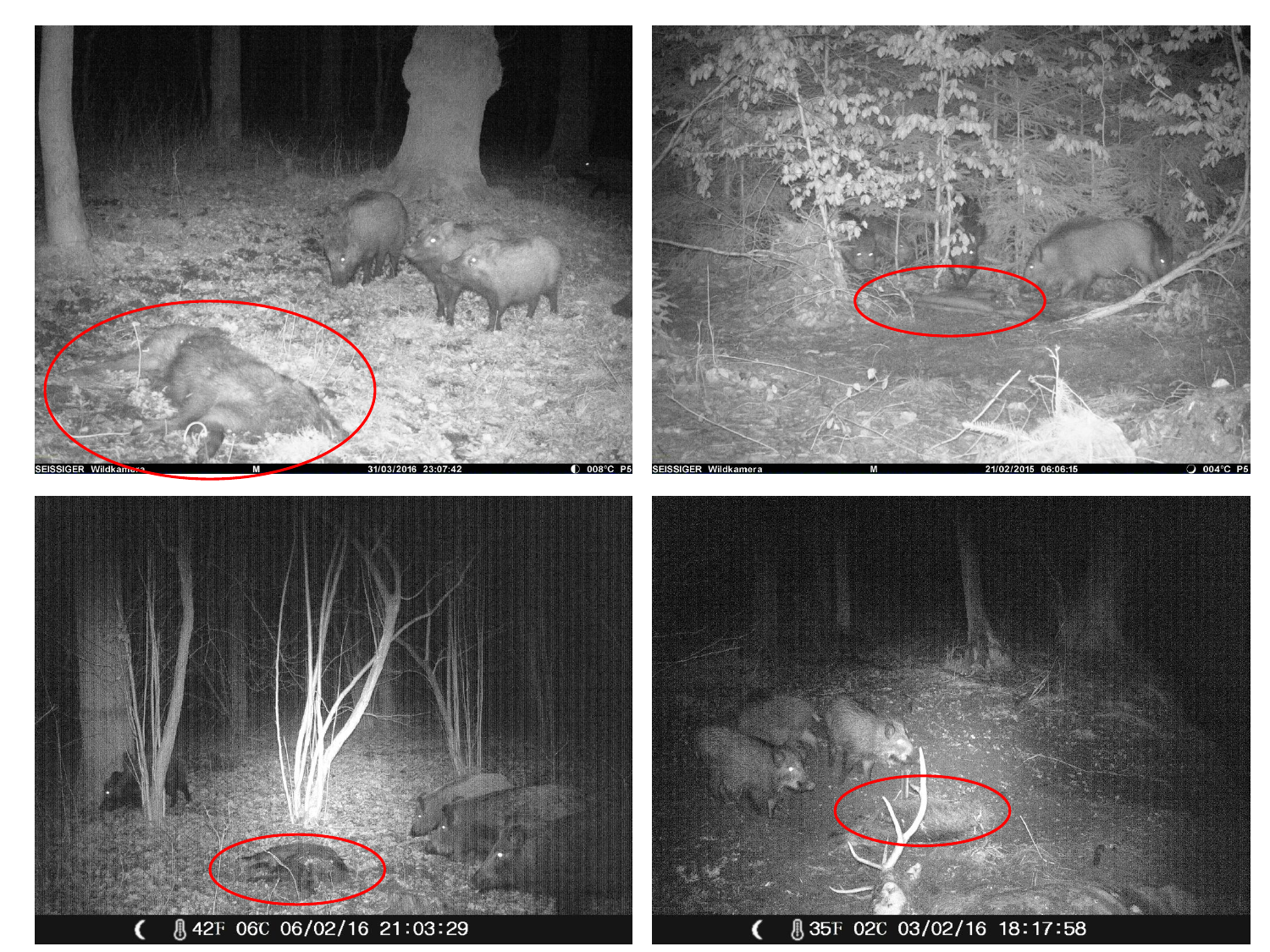

## Slide 2
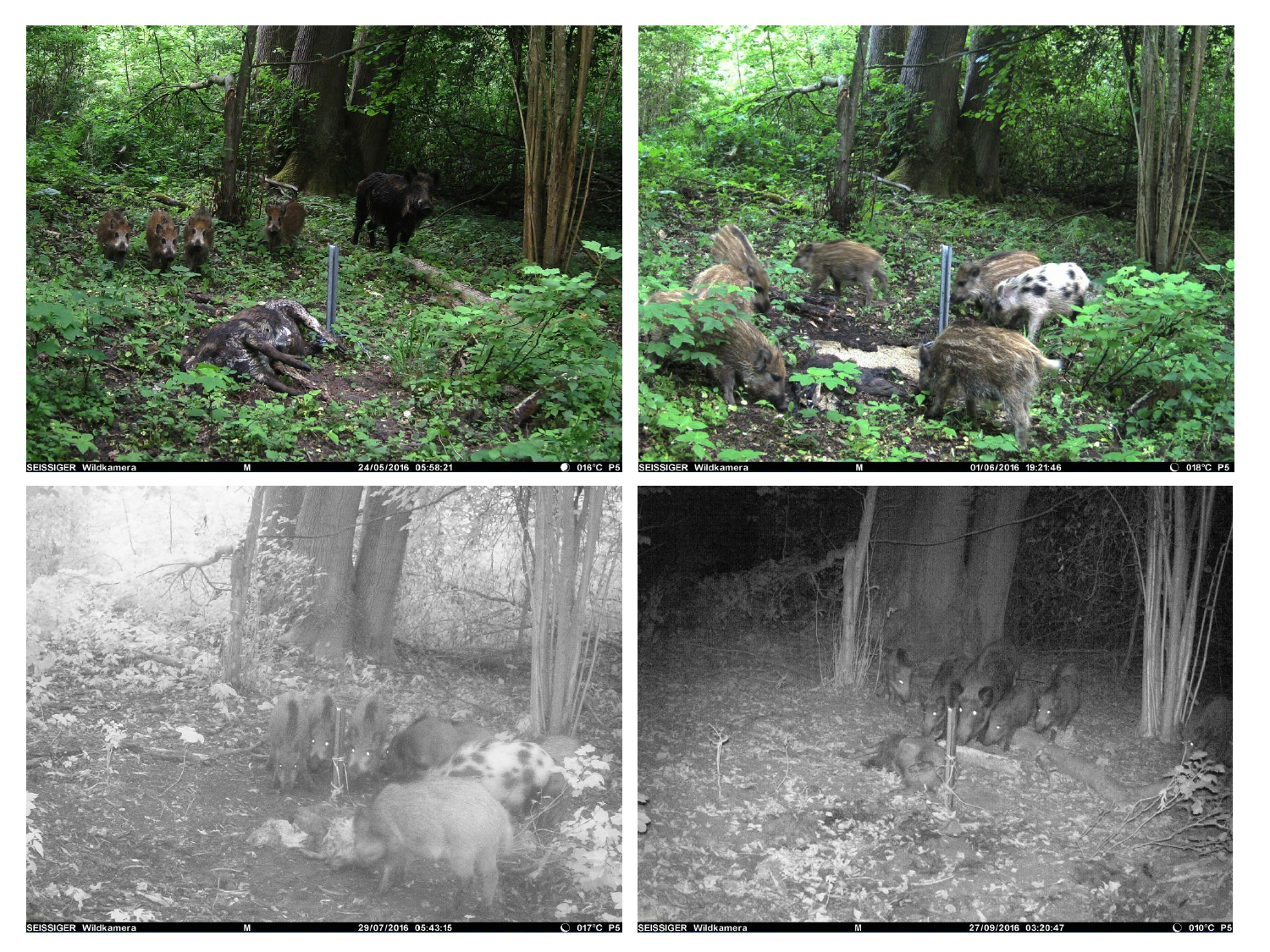

Supplement: ESM Figure 2 - Wild boar are curious, but do not touch the carcasses [file rsos170054supp3.pptx]

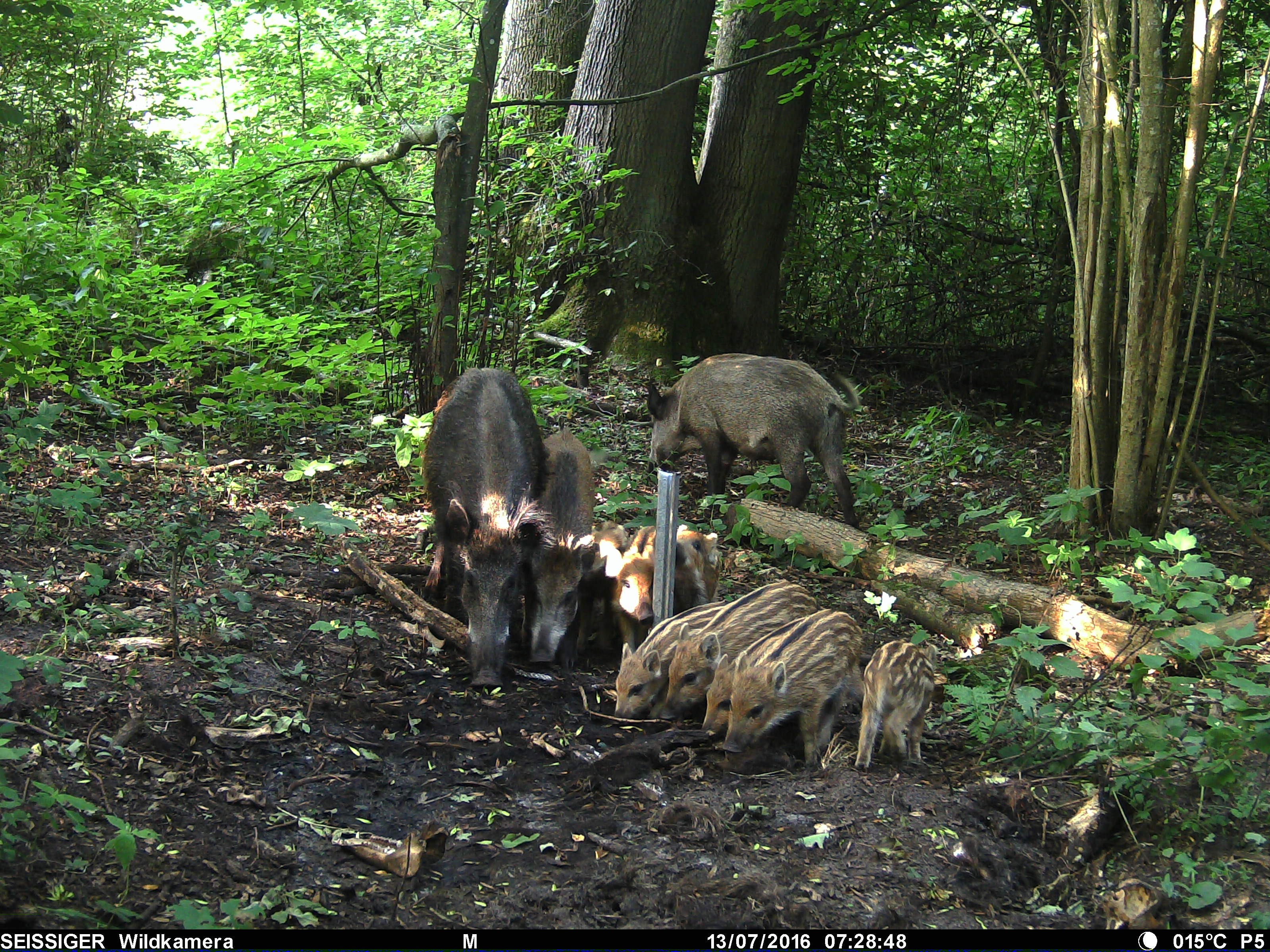

Supplement: ESM Figure 3 - Wild boar are attracted by the soft ground underneath rotten carcasses [file rsos170054supp4.jpg]

## Slide 1
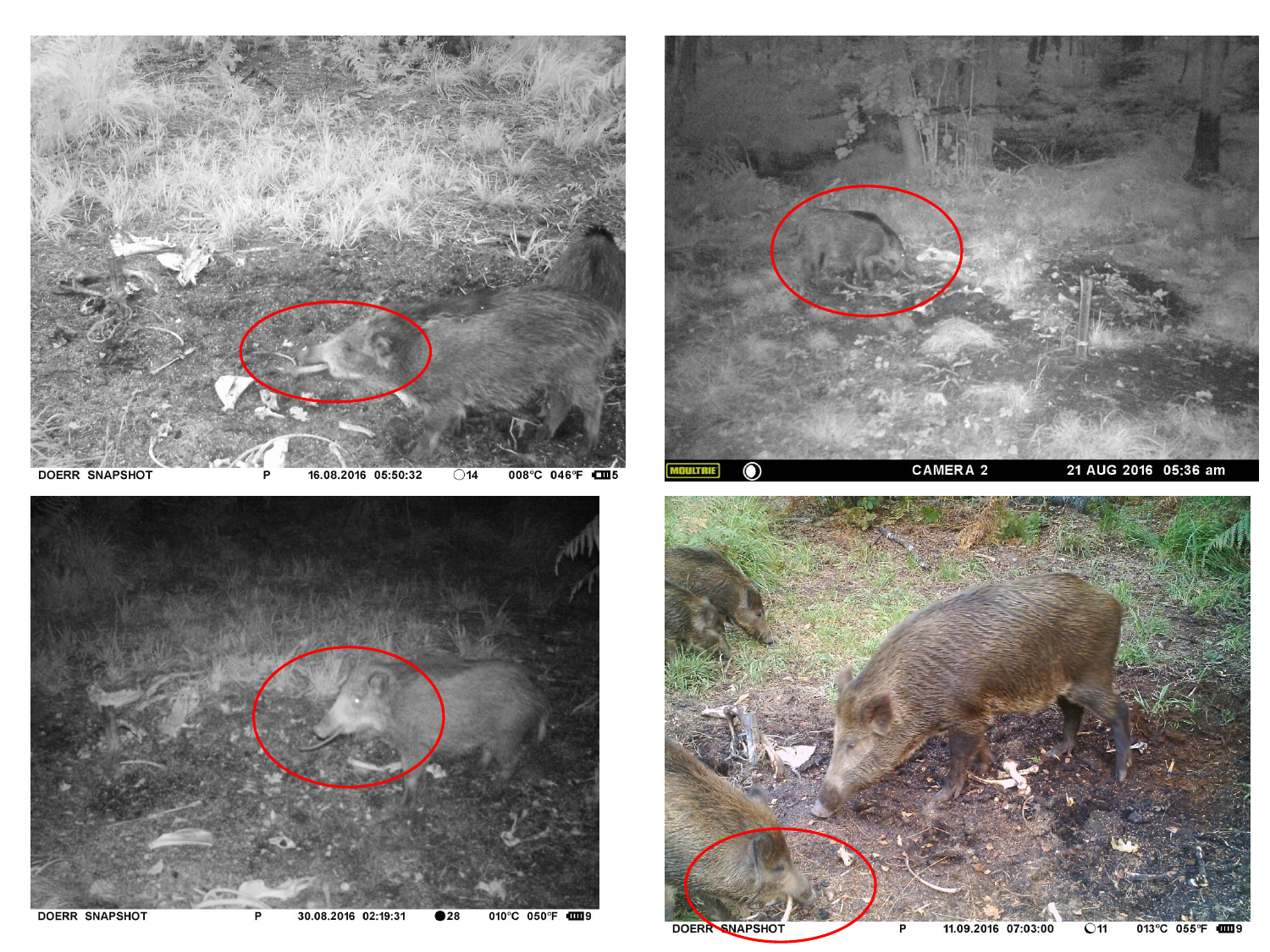

Supplement: ESM Figure 4 - Wild boar chewing on bones (bare ribs) after skeletonization is complete [file rsos170054supp5.pptx]

## Slide 1
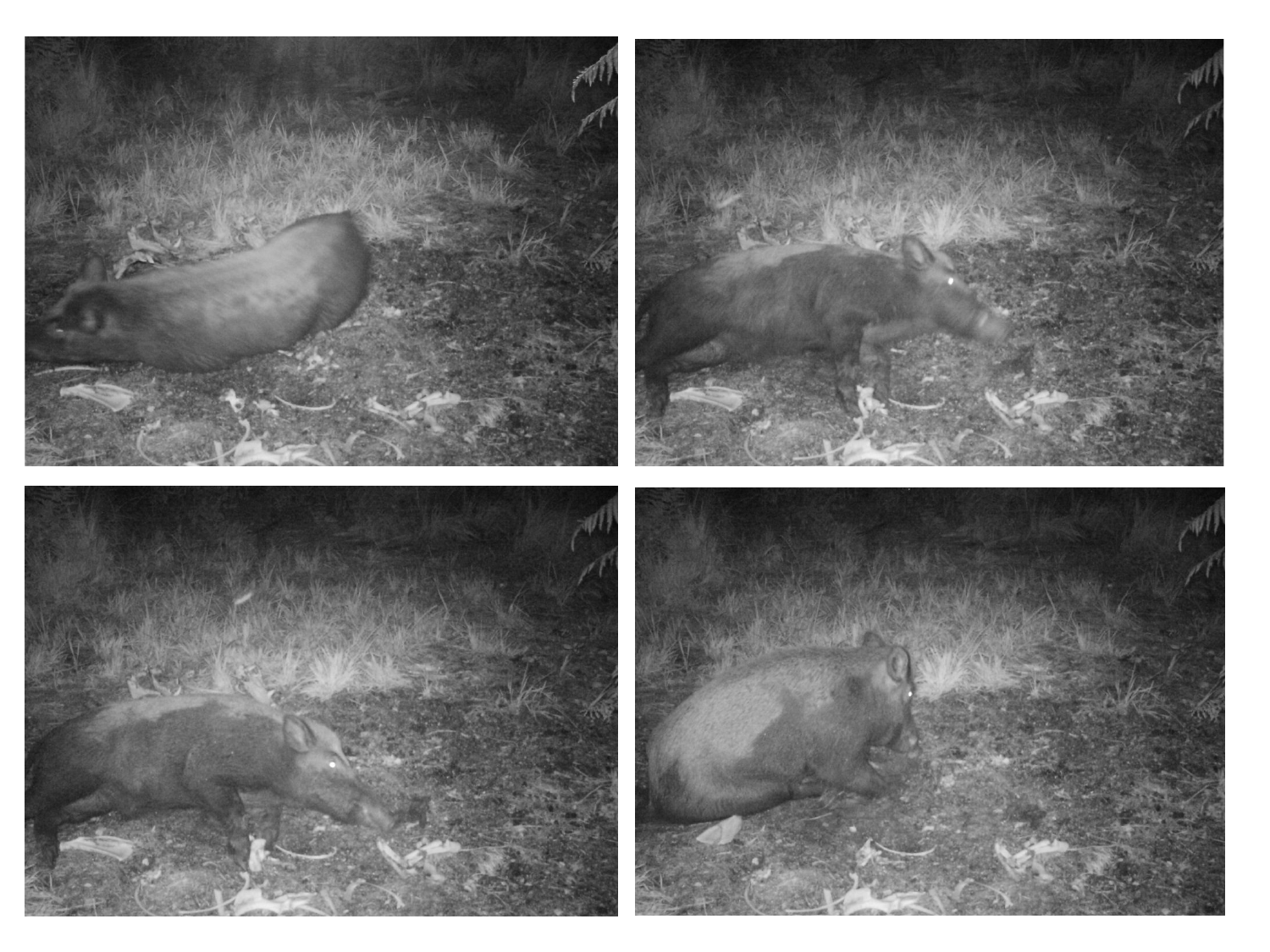

Supplement: ESM Figure 5 - Wild boar rolling on soft ground on site 3 [file rsos170054supp6.pptx]

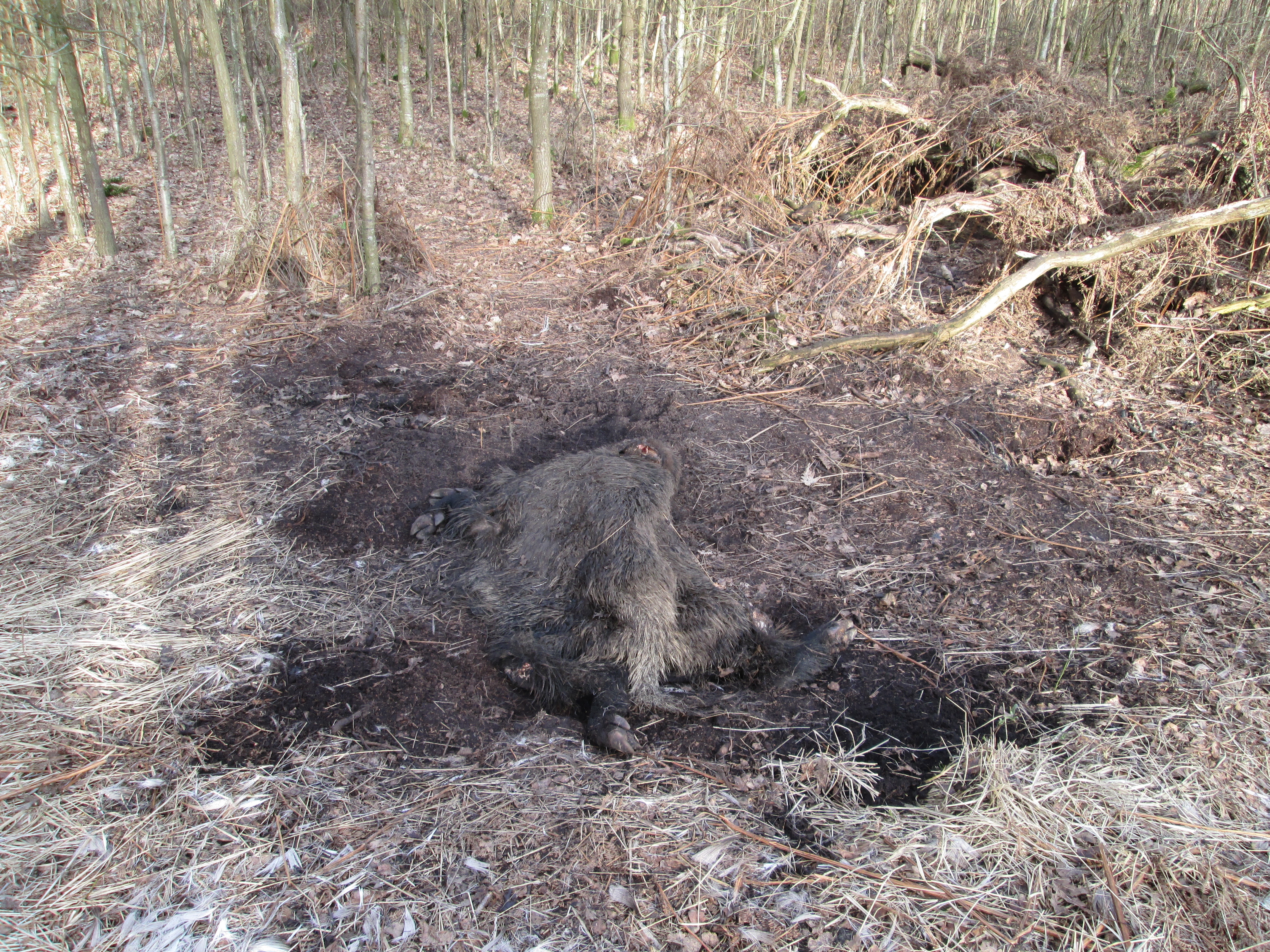

Supplement: ESM Figure 6 - Ground underneath carcass 3 is stirred up [file rsos170054supp7.jpg]

## Slide 1
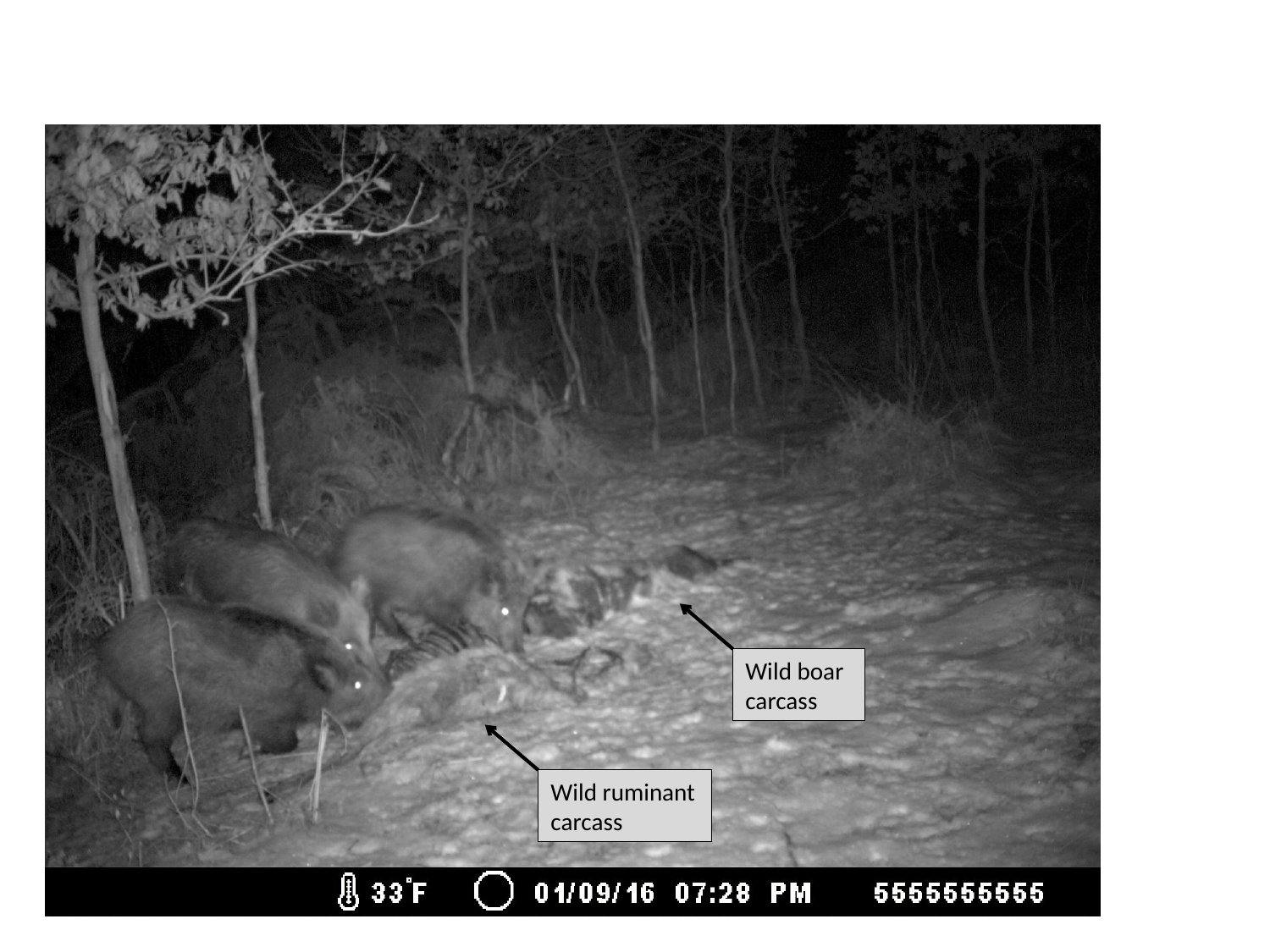

Wild boar carcass
Wild ruminant carcass

Supplement: ESM Figure 7 - Wild boar feeding on wild ruminant and leaving wild boar carcass aside [file rsos170054supp8.pptx]

## Slide 1
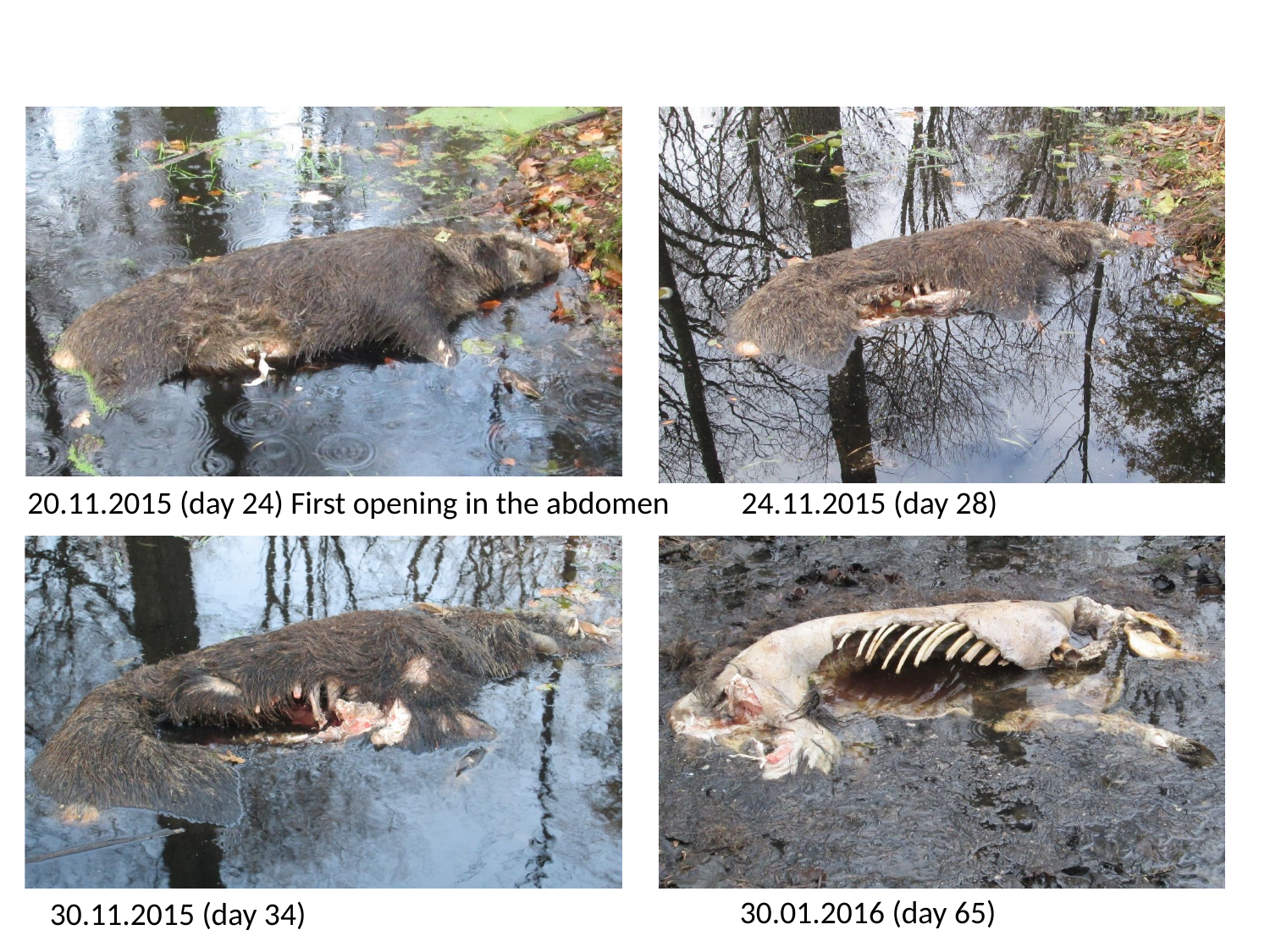

20.11.2015 (day 24) First opening in the abdomen
24.11.2015 (day 28)
30.01.2016 (day 65)
30.11.2015 (day 34)

Supplement: ESM Figure 8 - Skeletonization process of carcass 1 [file rsos170054supp9.pptx]
